# Long non‐coding RNA SNGH7 Is activated by SP1 and exerts oncogenic properties by interacting with EZH2 in ovarian cancer

**DOI:** 10.1111/jcmm.15373

**Published:** 2020-05-18

**Authors:** Zhuanli Bai, YinYing Wu, Shuheng Bai, Yanli Yan, Haojing Kang, Wen Ma, Jiangzhou Zhang, Ying Gao, Beina Hui, Hailin Ma, Rong Li, Xiaozhi Zhang, Juan Ren

**Affiliations:** ^1^ Department of Plastic and Aesthetic Maxillofacial Surgery First Affiliated Hospital of Xi'an Jiao Tong University Xi’an China; ^2^ Department of Chemotherapy, Oncology Department First Affiliated Hospital of Xi’an Jiaotong University Xi’an China; ^3^ Department of Radiotherapy, Oncology Department First Affiliated Hospital of Xi’an Jiaotong University Xi’an China; ^4^ Medical School Xi’an Jiaotong University Xi’an Xi’an China

**Keywords:** cell migration, EZH2, KLF2, lncRNA SHNG7, ovarian cancer

## Abstract

Long non‐coding RNAs (lncRNAs) are key regulators or a range of diseases and chronic conditions such as cancers, but how they function in the context of ovarian cancer (OC) is poorly understood. The Coding‐Potential Assessment Tool was used to assess the likely protein‐coding potential of SNHG7. SNHG7 expression was elevated in ovarian tumour tissues measured by qRT‐PCR. The online database JASPAR was used to predict the transcription factors binding to SNHG7. Twenty‐four‐well Transwell plates were used for invasion assays. RNA immunoprecipitation was performed to determine RNA‐protein associations. EdU assay was introduced to detect cell proliferation. Chromatin immunoprecipitation was performed to confirm the directly interaction between DNA and protein. We discovered that in the context of OC there is a significant up‐regulation of the lncRNA SNHG7. Knocking down this lncRNA disrupted both OC cell invasion and proliferation, while its overexpression had the opposite effect. SP1 binding sites were present in the SNHG7 promoter, and chromatin immunoprecipitation (ChIP) confirmed direct SP1 binding to this region, activating SNHG7 transcription. We found that at a mechanistic level in OC cells, KLF2 is a probable SNHG7 target, as we found that SHNCCC16 directly interacts with EZH2 and thus represses KLF2 expression. In summary, this research demonstrates that lncRNA SNHG7 is an SP1‐activated molecule that contributes to OC progression by providing a scaffold whereby EZH2 can repress KLF2 expression.

## 1 INTRODUCTION

Ovarian cancer (OC) is among a leading type of cancer that is diagnosed in women throughout the world, and it is the most dangerous cancer associated with the reproductive system in women.^1^ Numerous factors at both the environmental and genetic levels contribute to the risk of developing OC. The hormone oestrogen, for example, can drive tumour development through promotion of cellular proliferation and subsequently enhancing invasion.^2^ While key factors relevant to the development of this disease have been pinpointed, the multifactorial and complex nature of OC means that many aspects of its progression are still poorly understood, making it essential that the underlying molecular mechanisms governing OC be studied further.^3^

Recent work has established that more than three‐quarters of the human genome can transcribe non‐coding RNA molecules, the diversity of which is only beginning to be appreciated.^4^ As understanding of how these non‐coding RNAs function has improved, novel insights into the molecular basis of gene regulation have been gained, although some of these studies remain controversial. Long non‐coding RNAs (lncRNAs) are a recently identified subset of non‐coding RNAs that have been implicated in many diseases.^5^ Indeed, lncRNAs are thought to regulate onocogenes and tumour suppressor genes, thereby modulating carcinogenesis and serving as potential therapeutic markers of cancer progression.^6^, ^7^, ^8^ Many genes are commonly dysregulated in the context of OC, including PTENP1, UCA1 and NEAT1.^9^, ^10^, ^11^ To date, the extent of lncRNA research in the context of OC remains fairly limited, but the number of studies relating to this topic continues to grow. In the present study, we highlight a role for lncRNA SNHG7 and discuss other lncRNAs relevant to OC progression.

The mechanisms by which lncRNA execute their regulatory effects are complex and vary based on the specific target of study. For example, some drive direct signalling, whereas others may serve as decoys or even scaffolds that facilitate signalling pathways.^9^, ^12^, ^13^ KLF2 a key tumour suppressor gene of the Kruppel‐like factor (KLF) family, altered expression of which is associated with the progression of numerous forms of cancer.^14^, ^15^ As a tumour suppressor, KLF2 suppresses KRAF‐mediated tumour proliferation.^15^, ^16^ EZH2 is a factor that can counteract the tumour‐suppressive qualities of KLF2 by binding to its promoter region, silencing KLF2 transcription.^16^

In the present study, we assessed the expression, function and targets of lncRNA SNHG7 in the context of OC. We found that lncRNA SNHG7 was up‐regulated significantly within OC tissues. Knocking down lncRNA SNHG7 led to suppression of the growth and invasive potential of OC cells, suggesting that its expression is oncogenic. We found that in OC cells SP1 directly activated lncRNA SNHG7 transcription, and that this lncRNA in turn bound to EZH2, potentially allowing it to serve as a scaffold that leads to repression of KLF2 expression. In summary, that lncRNA SNHG7 may be a key regulator of OC progression and a viable target for future therapeutic intervention.

## 2 MATERIAL AND METHODS

### 2.1 Tissue collection

Thirty patients with OC who had been treated at the First Affiliated Hospital of Xi'an Jiaotong University were enrolled in this study. Liquid nitrogen was used for snap freezing of patient tissue samples, which were stored until use. All patients provided informed written consent, and this study was overseen by the Xi'an Jiaotong University Research Ethics Committee.

### 2.2 QRT‐PCR

TRIzol (Invitrogen, Carlsbad, CA, USA) allowed for RNA extraction from all samples based on manufacturer's protocols. cDNA was generated with the Reverse Transcription Kit (Takara, Dalian, China). ABI 7500 was used to conduct all quantitative real‐time PCR (qRT‐PCR) assays. GAPDH expression was used to normalize RNA amounts across samples. Table S1 lists all primers.

### 2.3 Cell culture

The Chinese Academy of Sciences cell bank was the source of all human OC A2780, OCC1, H8710 and SK‐OV3 lines and the control HMEC‐1 lines used. Cells were grown in RPMI 1640 Medium (Invitrogen) containing 10% foetal bovine serum, with penicillin/streptomycin at 37°C. Antibodies against N‐cadherin, E‐cadherin, SNAIL, Vimentin, GAPDH and secondary HRP‐conjugated antimouse IgG antibody were from Abcam (Cambridge, MA, USA).

### 2.4 Cell transfection

Overian Cancer cells were cultured overnight in 6‐well plates and were transfected with 100 nM of a specific siRNA or control via Lipofectamine 2000 (Invitrogen). Cells were then collected at appropriate time points for analysis. Table S1 lists siRNA sequences.

### 2.5 Edu assays

Twenty‐four hours after transfection, cell media was exchanged with fresh media. After an additional 28 hour, media was replaced with fresh media supplemented with 5 μm EdU (Life Technologies) and incubated for a further 20 hours. Four percent paraformaldehyde was then used to fix cells prior to immunofluorescent examination.

### 2.6 Cell migration and invasion assays

Twenty‐four‐well Transwell plates were used for invasion assays, with a 8‐μm sized pore (Corning, Corning, NY, USA). To the top chamber, 1 × 10^5^ cells in serum‐free media were added after it had been coated in Matrigel (BD, Franklin Lakes, NJ, USA). To the lower chamber, RPMI‐1640 supplemented with 10% FBS was added. After 24 hour, remaining cells in the upper chamber were discarded, and those in the lower chamber were stained with 0.5% Crystal violet after fixation. Five fields chosen at random were counted.

### 2.7 Scratch‐wound healing assay

Cells were grown in monolayers and wounded with a pipette tip. Detached cells were washed off with PBS, and new medium was added. Migration of cells was measured at 24 h as reduction of the wound area in each photographed field. At least five fields were photographed for each condition each time, and the gap was calculated using ImageJ software (version 1.44V, National Institutes of Health, Bethesda, MD, USA).

### 2.8 Plasmids construction and luciferase assays

SNHG7 promoter region (P4) containing the SP1 binding sites was PCR amplified from genomic DNA using PrimeSTAR HS DNA polymerase (Takara, Japan) with the primers 5'‐ CGGCTAGCGTAGAGCCAGCTCCGGCTACAAC −3' (forward) and 5'‐ CCCTCGAGGACGAGAAGGGAAGCGCCGCGCCT‐3' (reverse). P3 promoter was inserted with the primers 5'‐ CGGCTAGCGTAGAG CCAGCTCCGGC TACAAC −3' (forward) and 5'‐ CCCTCGAGCCGGGC AGCATGGGGCTCCCGCGA‐3' (reverse), P2 promoter with the primers 5'‐ CGGCTAGCGTAGAGCCAGCTCCGGCTACAAC −3' (forward) and 5'‐ CCCTCGAGTTAGGCC TGGAGGGGACACCAGGA‐3' (reverse), P1 promoter with the primers 5'‐ CGGCTAGCG TAGAGCCAGCTCCGGCTACAAC −3' (forward) and 5'‐ CCCTCGAGCCTAGCTCTGGATATGG TGCTAGA‐3' (reverse), and inserted into the Nhe I and XhoI sites of the pGL3 basic luciferase vector, respectively. 48‐hours post‐transfection cells were lysed with passive lysis buffer (Promega, Madison, WI, USA). 5 μL cell lysate was combined with 30 μL luciferase assay reagent from Promega, and a luminometer was used to read luciferase activity, with renilla serving as an internal transfection efficiency control.

### 2.9 RNA immunoprecipitation (RIP)

The EZMagna RIP kit (Millipore, Billerica, MA, USA) was purchased, and complete RIP lysis buffer was used to lyse SK‐OV3 cells, and this lysate was then combined with control or antibody‐conjugated magnetic beads against EZH2, KDM1A and SUZ12 (Millipore, Billerica, MA, USA) for 6 hours at 4°C. Beads were washed, and Proteinase K was then used to eliminate proteins. qRT‐PCR was then performed on purified RNA to assess the presence of lncRNA SNHG7.

### 2.10 Chromatin immunoprecipitation (chip)

Cross‐linking of OC cells was conducted for 10 minutes using formaldehyde. Cell lysates were then sonicated to produce 200‐300 bp chromatin fragments, which were then immunoprecipitated with SP1, H3K27me3, EZH2 or control IgG. Precipitated DNA was analysed by qRT‐PCR. Table S1 lists primer sequences.

### 2.11 Western blotting

RIPA buffer was used for cell lysis. Bio‐Rad protein assay system (Bio‐Rad Laboratories, Richmond, CA, USA) was used for protein quantification. Thirty μg of each sample were run on a 12% SDS–PAGE gel before transfer to a 0.22 mm nitrocellulose membrane (Osmonics, Westborough, MA, USA). Five percent non‐fat milk powder in TBS‐T was used for blocking for 1h at room temperature, and blots were then incubated for 1 h at room temperature (RT) with blocking buffer containing a 1:1000 dilution of either anti‐E‐cadherin, N‐cadherin, SNAIL, Vimentin or GAPDH. Blots were washed with TBS‐, incubated for 1 h at RT in blocking buffer with a 1:10,000 secondary HRP‐conjugated anti‐rabbit Ig (Amersham, Arlington Heights, IL, USA). Blots were washed again and developed with ECL reagents based on manufacturer's recommendation. GAPDH was used to normalize protein expression.

### 2.12 Tumour studies

Athymic nude BALB/c mice from the Shanghai Experimental Animal Center were kepy based on the guidelines of the Animal Research Committee of First Affiliated Hospital of Xi'an Jiaotong Universit. Mice received a dorsal flank injection of 5 × 10^6^ SK‐OV3 cells on the left side (four mice per group), and tumour growth was assessed daily. Tumour volumes were determined according to tumour size = *ab^2^*/2*,* with *a* being the larger and *b* the smaller of measured dimensions. Twenty‐two days after injection with tumour cells, mice were euthanized and tumours extracted. Tumour weight was measured.

### 2.13 Immunohistochemistry (IHC)

Samples of xenograft tumour tissue underwent Ki67 and H&E staining, using anti‐Ki67 (Abcam, Cambridge, MA, USA). A pathologist and the author independently scored the results from IHC stains.

### 2.14 Statistical analysis

Results are expressed as mean ± SD. All experiments were performed in triplicate at a minimum. Two‐tailed Student's t tests and Wilcoxon rank sum test were used for statistical analyses. The threshold for statistical significance was *P* < .05. *P < .05; **P < .01.

## 3 RESULTS

### 3.1 SNHG7 was a oncogenic lncRNA and up‐regulated in OC

**Figure 1:**
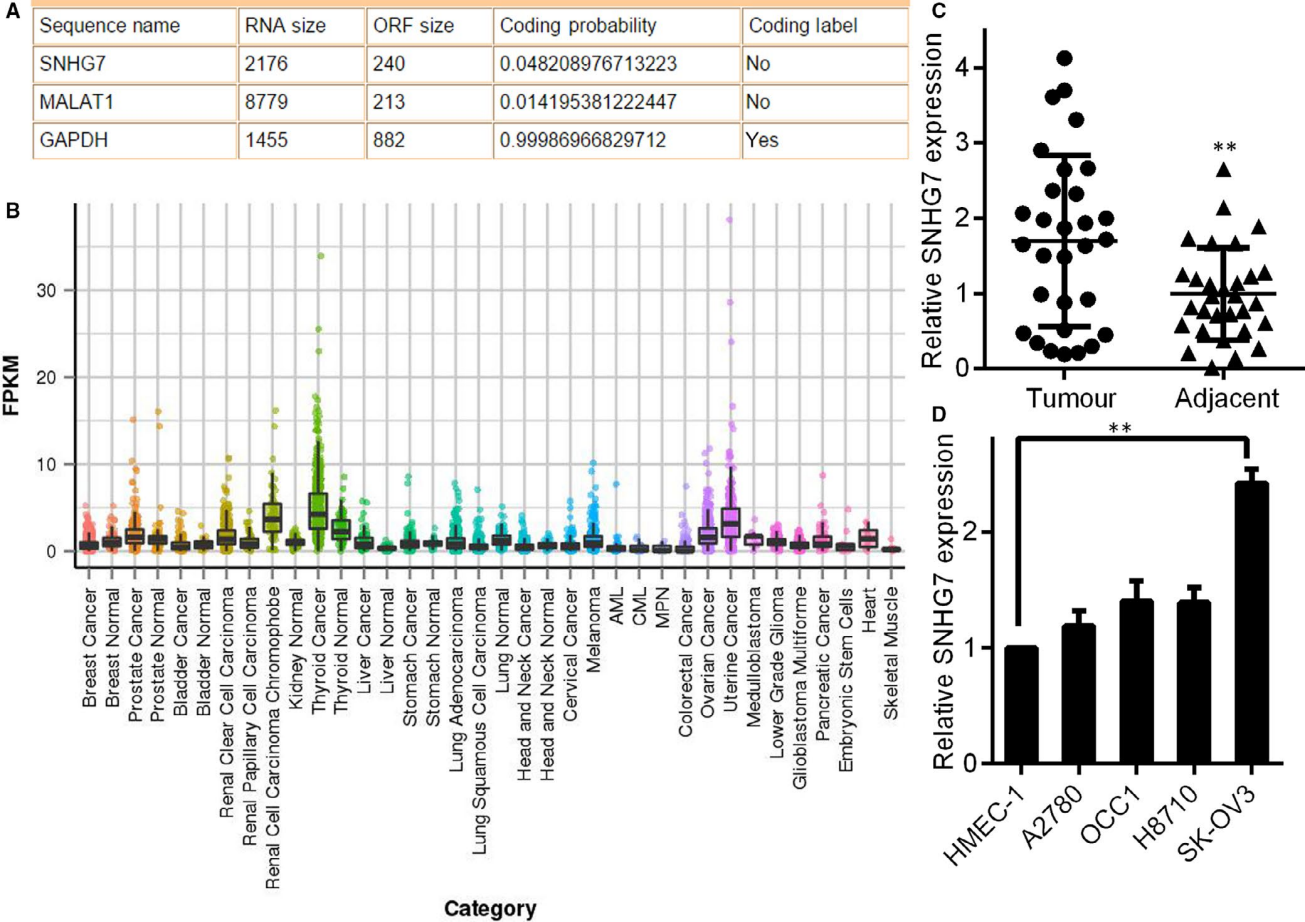
SNHG7 is up‐regulated in the context of OC. (A) Coding‐Potential Assessment Tool predictions regarding the SNHG7, MALAT1 and GAPDH sequences. (B) SNHG7 expression across cancer and normal tissue types as derived from the MiTranscriptome database. Expression is indicated by the fragments per kilobase of exon per million fragments mapped algorithm. (C) qRT‐PCR measurement of SNHG7 expression in 30 cancerous and 30 normal ovarian tissues, normalized to GAPDH. D. Relative levels of SNHG7 in the HMEC‐1, A2780, H8710, SK‐OV3 and OCC1 cell lines. **P < .01

To determine whether SNHG7 was truly a lncRNA, the likely protein‐coding potential of this gene was assessed with the Coding‐Potential Assessment Tool.^17^ This assessment determined that SNHG7 was a lncRNA with a less than 5% chance of coding for a protein (Figure 1A). Comparison with the MiTranscriptome database demonstrated SNHG7 up‐regulation in a number of cancers types (Figure 1B).^18^ Importantly, SNHG7 expression was elevated in ovarian tumour tissues as measured by qRT‐PCR compared to control non‐cancerous tissues, indicating that SNHG7 is significantly up‐regulated during OC (*P* < .01, Figure 1C). We assessed SNHG7 expression in a normal control cell lines: HMEC‐1 and multiple OC cell lines: A2780, H8710, SK‐OV3 and OCC1. SNHG7 was up‐regulated in all of these cell lines relative to normal cell line controls (Figure 1D). Together, these findings suggest SNHG7 regulation is altered in tumours and that there may be a functional role for this lncRNA in cancer.

**Figure 2:**
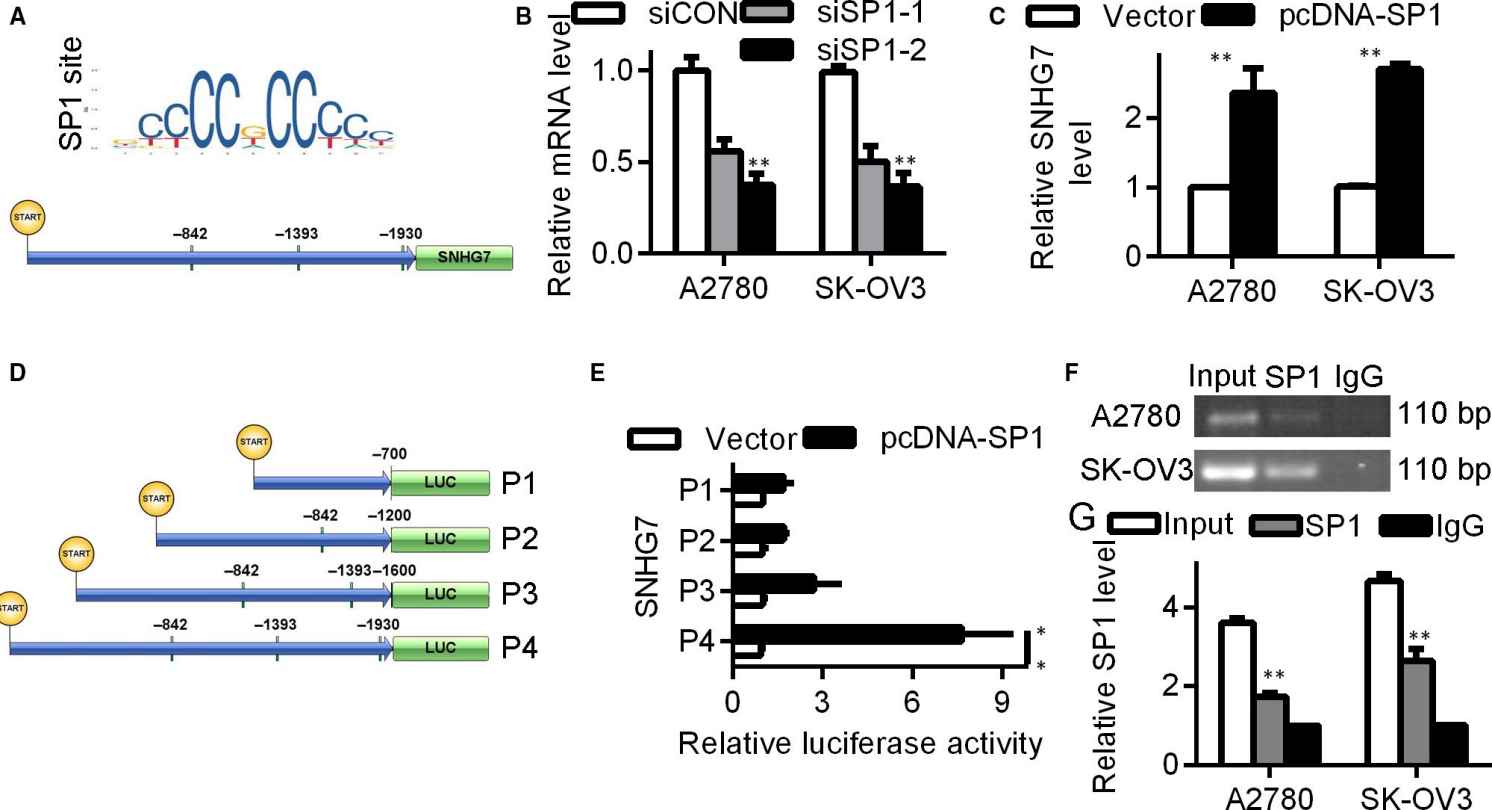
SP1 binds the SNHG7 promoter, inducing transcription. (A) JASPAR prediction of an SP1 binding site in the SNHG7 promoter. (B) SNHG7 expression in A2780 and SK‐OV3 cells after knock‐down of SP1. (C) SNHG7 expression in A2780 and SK‐OV3 cells overexpressing SP1. (D) Luciferase reporter construction vector. (E) Assessment of the SNHG7 promoter SP1 binding site via dual luciferase reporter assay. (F and G) ChIP assessment of SP1 binding to the SNHG7 promoter, revealing significantly greater SNHG7 levels in anti‐SP1 samples than controls. Input served as positive control and IgG as negative control. *P < .05, **P < .01

Multiple transcription factors including E2F1 and SP1 are known to regulate the transcription of lncRNAs.^19^ The online database JASPAR was used to predict the transcription factors binding to SNHG7, and SP1 was highly predicted to bind to this lncRNA (Figure 2A). SNHG7 expression was down‐regulated upon siRNA‐mediated SP1 silencing (Figure 2B). Conversely, overexpression of SP1 enhanced SNHG7 expression (Figure 2C). ChIP confirmed SP1 binding to the promoter region of SNHG7 near the P4 region (Figure 2D and 2E). Luciferase reporter assays also confirmed SP1 binding to this (−1930 bp) binding site, but not to other tested sites (Figure 2F and 2G).

### 3.2 Knock‐down of SNHG7 inhibits in vitro cell growth, migration and invasion

**Figure 3:**
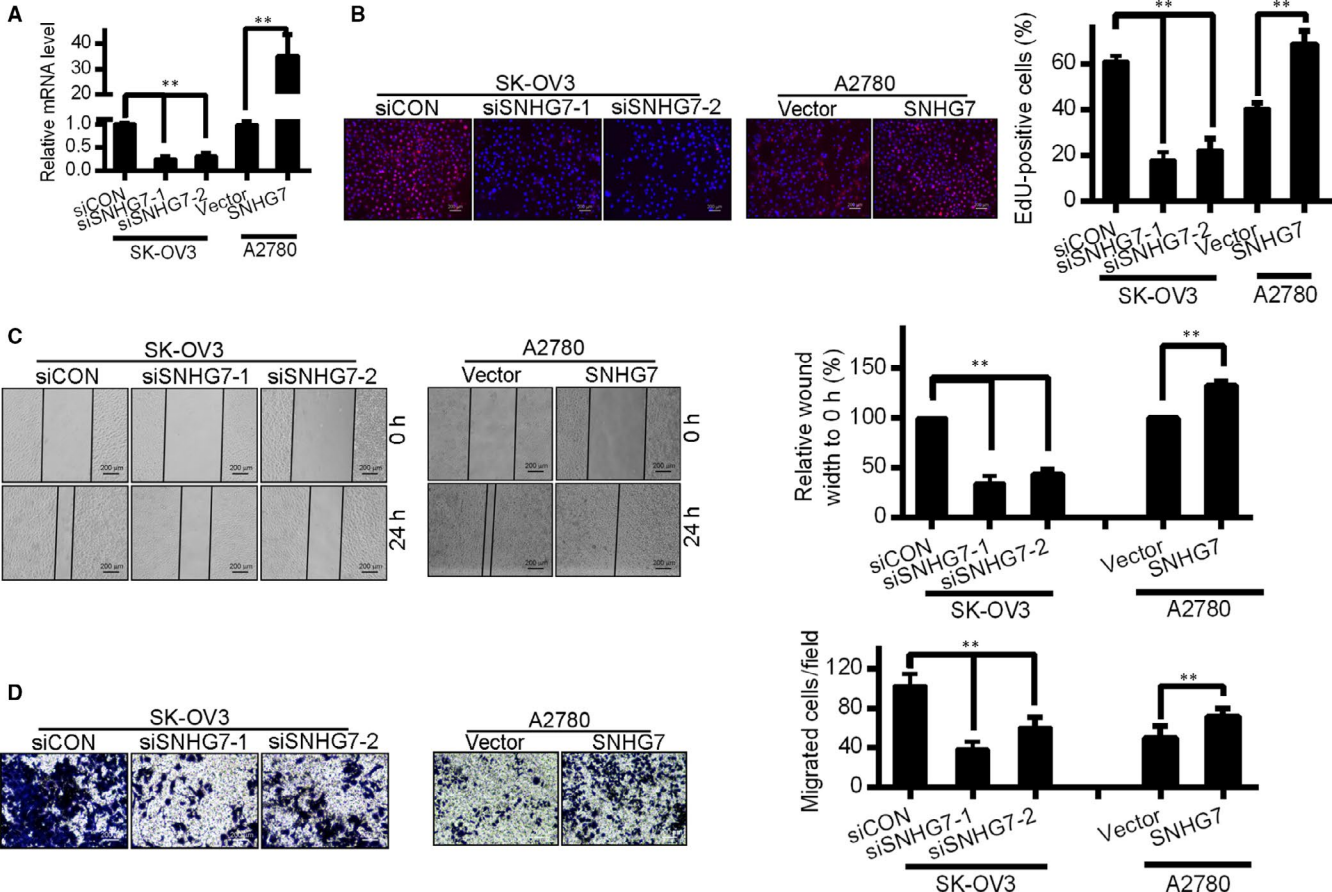
Knock‐down of SNHG7 inhibits OC cell growth, migration and invasion in vitro. (A) Relative mRNA levels in SK‐OV3 and A2780 cells measured by qRT‐PCR after knock‐down or overexpression of SNHG7. (B) Cell proliferation was determined by EdU assay in SK‐OV3 and A2780 cells. Bar = 200 μm. (C) Wound healing assay during a period of 24 h after knock‐down or overexpression of SNHG7 in OC cells. Bar = 200 μm. (D) Transwell invasion assays showed the invasive potential of SK‐OV3 and A2780 cells following suppression or overexpression of lncRNA SNHG7. *P < .05, **P < .01

We next sought to assess whether suppressing SNHG7 in A2780 and SK‐OV3 OC cells would affect their biology. SNHG7 was down‐regulated in SK‐OV3 cells and up‐regulated in A2780 cells. For knock‐down, two SNHG7 targeting siRNAs were used. Fourty‐eight hours after transfection, RT‐qPCR confirmed a significant decrease in SNHG7 expression, and the most efficient siRNAs si‐SNHG7‐1 and si‐SNHG7‐2 were used for all following in vitro experiments (Figure 3A). To assess the functional relevance of SNHG7 in OC cells, we assessed the proliferation and migration of cells SNHG7 knock‐down or—overexpressing cells via 5‐ethynyl‐2′‐deoxyuridine (EDU), wound healing and Transwell assays. The frequency of EDU‐positive cells confirmed cell growth was significantly impaired by SNHG7 knock‐down, while its overexpression was associated with increased growth (Figure 3B). Wound healing assays demonstrated lower rates of migration in SNHG7 knock‐down cells relative to controls, whereas overexpression increased this in A2780 cells (Figure 3C). Matrigel‐coated Transwell chambers were used for invasion assays, which demonstrated that lncRNA SNHG7 silencing was linked to a marked reduction in the invasive potential of SK‐OV3 cells, while its overexpression enhanced A2780 cell invasion (Figure 3D). These experiments suggest that disrupting SNHG7 expression can in turn disrupt malignant phenotypes in OC cells, whereas its overexpression enhances these phenotypes—a finding consistent with clinical results.

### 3.3 Inhibiting SNHG7 expression impairs metastatic properties by reversing EMT in oc cells

**Figure 4:**
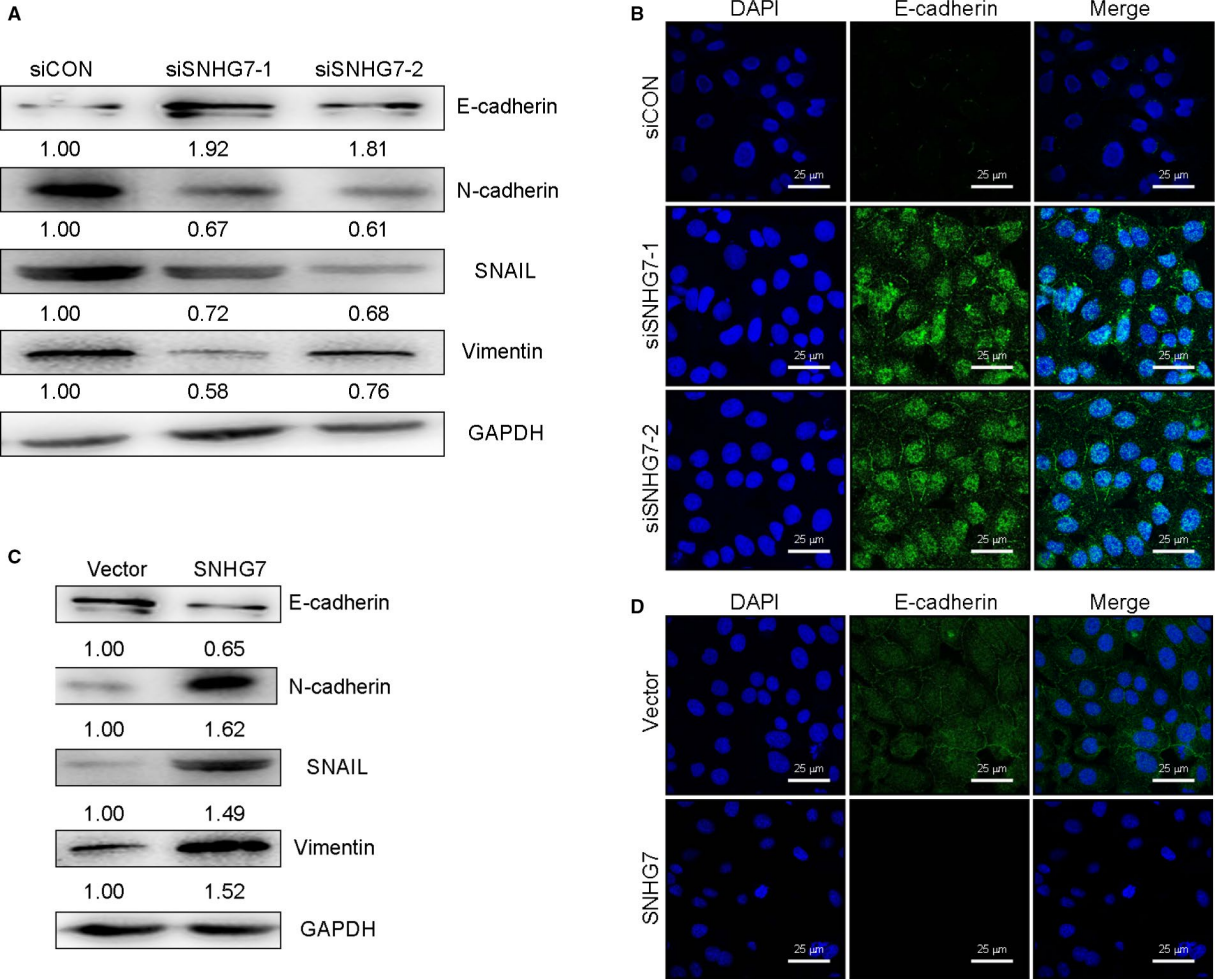
SNHG7 promotes metastasis via EMT reversal (A) Western blot and (B) immunofluorescence detection of epithelial (E‐cadherin) and mesenchymal (N‐cadherin, Vimentin, and SNAIL) markers in SK‐OV3 cells transfected with siCON, siSNHG7‐1 or siSNHG7‐2. (C) Western blot and (D) immunofluorescence detection of epithelial/mesenchymal markers in A2780 cells transfected with SNHG7 or control Vectors, respectively

Similarly to the above results, SK‐OV3 in which SNHG7 was silenced had significantly reduced wound healing capabilities (Figure 4A). Migration and invasive potential of these cells was similarly reduced (Figure 4B and 4C).

Migration and invasion are cornerstones of cancer malignancy and spread, and the Epithelial‐mesenchymal transition (EMT) is closely linked to such invasive potential. To assess whether SNHG7 was connected with EMT in OC cells, EMT protein was assessed in cells after SNHG7 knock‐down or overexpression by Western blotting and immunofluorescent analysis. SNHG7 down‐regulation was linked with elevated epithelial marker (E‐cadherin) and mesenchymal marker (N‐cadherin, SNAIL and Vimentin) expression in SK‐OV3 cells (Figure 3A and 3B). In contrast, SNHG7 reduced expression levels of these proteins in A2780 cells (Figure 3C and 3D). Therefore, SNHG7 is associated with EMT induction in OC cells, enhancing migration and invasion.

### 3.4 SNHG7 inhibits KLF2 VIA EZH2 binding

**Figure 5:**
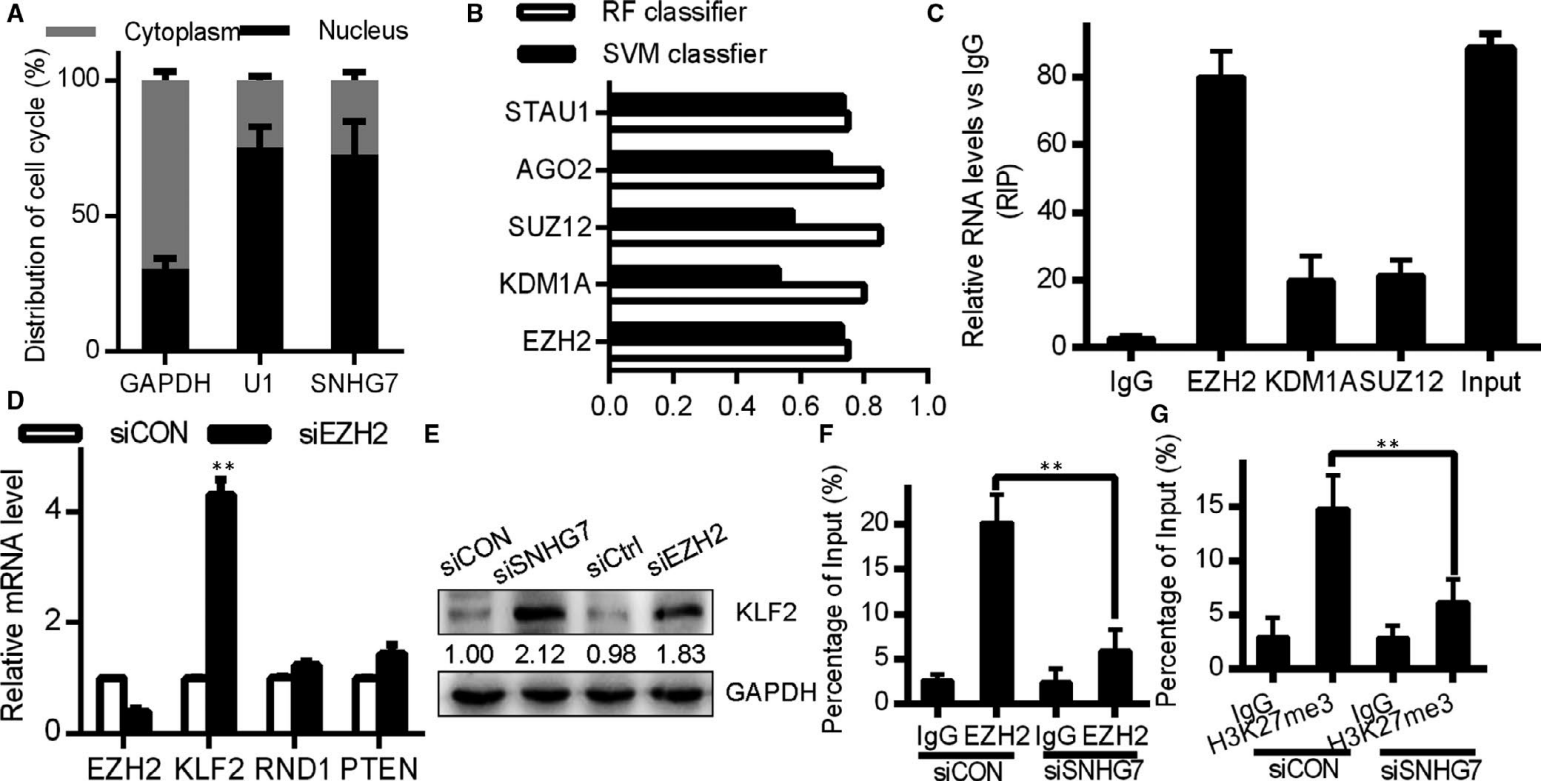
SNHG7 silences KLF2 transcription by epigenetic mechanisms via EZH2 binding. (A) Nuclear and cytoplasmic SNHG7 levels in SK‐OV3 cells as measured by qRT‐PCR. (B) Prediction of the interaction probabilities between SNHG7 and RNA binding proteins using the database at http://pridb.gdcb.iastate.edu/RPISeq/. (C) RIP assays conducted in SK‐OV3 cells, with SNHG7 co‐precipitation assessed by qRT‐PCR. (D) EZH2, KLF2, RND1 and PTEN expression in SK‐OV3 cells after EZH2 knock‐down. (E) Western blots for KLF2 after SNHG7 or EZH2 knock‐down in SK‐OV3 cells. (F) and (G) ChIP‐qPCR for H3K27 trimethylation of the KLF2 promoter in SK‐OV3 cells after SNHG7 knock‐down. *P < .05, **P < .01

To determine how SNHG7 acts in OC cells, we assessed its subcellular localization and we found that it was a primarily nuclear lncRNA (Figure 5A). As such, we postulated that SNHG7 may control transcriptional and post‐transcriptional gene activities. An RNA‐protein interaction prediction (http://pridb.gdcb.iastate.edu/RPISeq/) was performed that suggested SNHG7 may bind to STAU1, AGO2, SUZ12, KDM1A and EZH2 (RF and SVM scores >0.5; Figure 5B). To assess these interactions, RIP analysis was performed that revealed a strong physical interaction between SNHG7 and EZH2 (Figure 5C). EZH2 is a nuclear protein associated with histone/DNA methylation, indicating that SNHG7 may alter target genes at the epigenetic level. We therefore selected EZH2 target genes with tumour‐suppressive functions (KLF2, RND1 or PTEN) that could be involved in the link between SNHG7 and OC progression. qRT‐PCR analysis revealed that SNHG7 knock‐down was associated with an increase in KLF2 expression, while there were no changes in any of the other genes (Figure 5D). Western blotting confirmed this finding at the protein level in SK‐OV3 cells (Figure 5E). H3K27 trimethylation by the PRC2 complex is linked to negative gene regulation.^20^ SNHG7 most likely constrains KLF2 expression via PRC2 complex recruitment to the KLF2 promoter, leading to proximal H3K27 trimethylation. Using three primer pairs spanning a 2000bp section of KLF2, we performed chromatin immunoprecipitation (ChIP) in SNHG7 knock‐down cells. These results revealed that SNHG7 knock‐down decreased EZH2 binding and H3K27me3 at the KLF2 promoter (Figure 5F and 5G). These results thus suggest that SNHG7 may suppress KLF2 expression via binding EZH2 (a key PRC2 component), ultimately driving OC development.

### 3.5 KLF2 suppression is linked with the oncogenic activity of SNHG7

**Figure 6:**
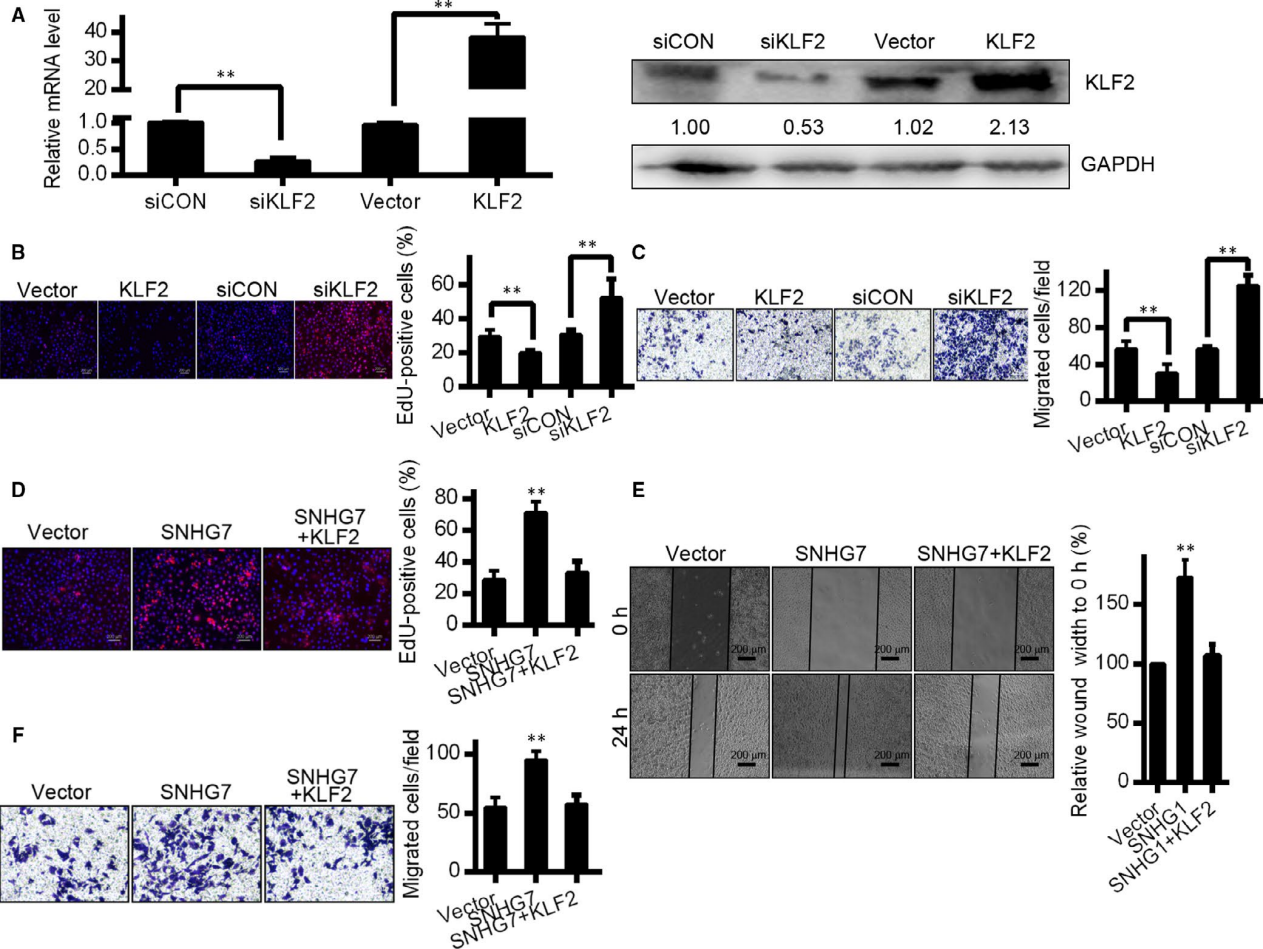
SNHG7 suppresses KLF2 expression. (A) KLF2 mRNA expression as assessed by qRT‐PCR and Western blot in SK‐OV3 cells were transfected with siCON, siKLF2, or overexpressing empty vector or pcDNA‐KLF2 vectors. (B) EdU staining assessment of SK‐OV3 proliferation after control or KLF2 siRNA transfection. (C) Transwell measurements using KLF2 or siKLF2‐transfected SK‐OV3 cells. (D) EdU staining measurements of SK‐OV3 cells proliferation after empty vector, SNHG7 or SNHG7 and KLF2 transfection. (E) Wound healing assessment of cell migration in SK‐OV3 with the same constructs as in (D). (F) Transwell assays to assess invasive potential of SK‐OV3 cells transfected with these same constructs. *P < .05, **P < .01

To confirm the relevance of KLF2 to OC cell migration/proliferation, KLF2 was either knocked down or overexpressed in SK‐OV3 cells (Figure 6A). Consistent with previous results, KLF2 knock‐down was linked to increased cell proliferation as assessed by EDU, and increased the invasive potential of these cells in a Transwell assay (Figure 6B and 6C). The effects of KLF2 expression are thus the opposite of those of SNHG7 in OC cells. In order to perform rescue experiments, SK‐OV3 cells were co‐transfected to overexpress both SNHG7 and KLF2. Proliferation of co‐transfected cells was enhanced relative to SK‐OV3 cells transfected with SNHG7 alone (Figure 6D). Migration and invasive potential were also rescued in these co‐transfected cells (Figure 6E and 6F). This reveals that the effects of SNHG7 in OC cells may be partly mediated via KLF2 repression.

### 3.6 Knock‐down of SNHG7 inhibits OC tumorigenesis in vivo

**Figure 7:**
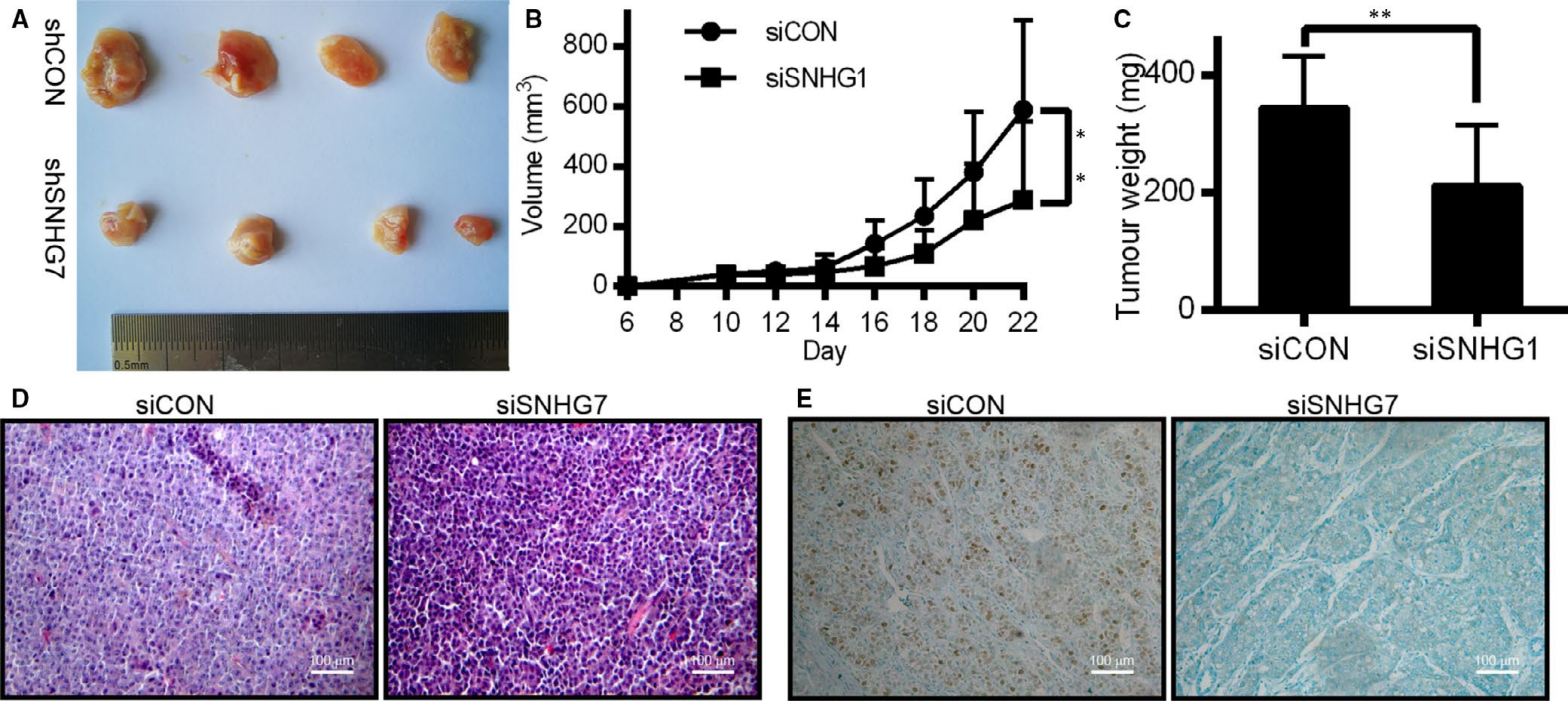
SNHG7 knock‐down inhibits OC tumorigenesis in vivo. (A) Tumour numbers after removal from mice. (B) Tumour volumes, calculated every 2 days after implantation. (C) Tumour weights at time of harvest (D) and (E) Representative images H&E and IHC stains of the tumour, suggesting decreased proliferation based on reduced Ki‐67. *P < .05 and **P < .01

To assess the relevance of SNHG7 to tumorigenesis in vivo, SK‐OV3 cells transfected with siSNHG7/siCON were injected into nude BALB/c mice. Consistent with our in vitro findings, tumour growth was significantly impaired in siSNHG7 mice relative to controls (Figure 7A and 7B). Twenty‐two days post‐inoculation, tumour weights were significantly lower in the siSNHG7 mice (Figure 7C). siSNHG7‐transfected cell‐derived tumours also had lower Ki67 expression levels based on IHC measurements (Figure 7D and 7E). SNHG7 levels were lower in the tumours from the siSNHG7 group (Figure 7E). These findings suggest that SNHG7 is significantly associated with OC proliferation in vivo.

## 4 DISCUSSION

Modern advances in sequencing technology have revealed that upwards of 97% of the human genome can be transcribed as non‐coding RNA molecules (ncRNAs). These ncRNAs are typically classified based on length into either short (<200 nt) or long (>200 nt) ncRNAs.^21^ Accumulating evidence suggests that long non‐coding RNAs can serve critical functions in the development and progression of cancer.^22^, ^23^, ^24^ The precise molecular mechanisms underlying these roles, however, remain to be fully explored.

In the present study, we have uncovered a novel for SNHG7 in OC cells. SNHG7 is markedly up‐regulated in the context of OC tissues relative to normal tissue controls. Knock‐down of SNHG7 decreased the proliferation, migration and invasive potential of OC cells, whereas its overexpression had the opposite effect. This was true both in vitro and in vivo in a mouse xenograft model. This suggests that SNHG7 may serve as an important clinical marker of OC prognosis in humans.

There are a diverse range of molecular mechanisms by which lncRNAs can exert biological effects relevant to cancer.^25^, ^26^, ^27^ One such key function occurs when lncRNAs serve as molecular scaffolds, indirectly affecting biology by alerting the activities of other proteins and signalling molecules. For example, a lncRNA known as HOTTIP can associate with the PRC2 and WDR5/MLL1 chromatin‐modifying complexes.^28^ Similarly, HOTAIR can directly interact with PRC2 and the LSD1/CoREST/REST complex.^29^

KLF2 is a key mediator of cancer development and tumour progression.^14^, ^30^ We found that SNHG7 is able to suppress KLF2 expression at both the protein and RNA level in OC cells.^14^ We further determined that SNHG7 is primarily located in the nucleus, suggesting a potential role for this lncRNA in mediating transcription.

We further performed the RIP assays which revealed that SNHG7 directly binds to EZH2, thereby silencing KLF2 expression. Chromatin immunoprecipitation analyses further revealed that EZH2 directly binds to the KLF2 promoter region, inducing H3K27 trimethylation. This indicated that SNHG7 drives OC cell proliferation by regulating KLF2 expression through EZH2 binding (Figure S1).

In summary, we determined that SNHG7 is up‐regulated in OC tissues and cell lines. SNHG7 serves as a potential oncogene by promoting cellular proliferation, migration and invasion. SNHG7 may induce KLF2 epigenetic silencing via EZH2 binding. Additional future study of SNHG7 may ultimately serve to enhance the utility of this biomarker for the diagnosis and treatment of OC in humans, reducing the spread of this deadly disease.

Our study revealed that the taken together, our results indicate that SNHG7 is significantly up‐regulated in ovarian cancer tissues. SNHG7 may exert oncogenic activity by epigenetically altering KLF2 transcription via binding to EZH2. Further studies of the clinical relevance and functionality of SNHG7 may play a key role in improving OC prognoses and diagnoses.

## 5 ETHICS APPROVAL AND CONSENT TO PARTICIPATE

This study was approved by the Ethics Committees of Xi'an Jiaotong University. Informed consent was obtained from each patient, including consent for their samples to be taken and used for research purposes before surgery. All in vivo protocols were approved by the Institutional Animal Care and Use Committee of Xi'an Jiaotong University.

Consent for publication: Not applicable.

## CONFLICT OF INTEREST

The authors confirm that there are no conflicts of interest.

## AUTHORS’ CONTRIBUTIONS

JR conceived and supervised the study; JR and ZLB designed experiments; JR, ZLB, YYW, SHB, YLY, HJK, WM, JZZ, YG and BNH performed experiments; HLM provided new tools and reagents; JR, HLM, XZZ and RL analysed data; JR and ZLB wrote the manuscript; JR and XZZ made manuscript revisions. All authors have read and approved the final version of this submission.

## Supporting information

Fig S1

Table S1

## Data Availability

The data sets used and analysed during the current study are available from the MiTranscriptome database (www.mitranscriptome.org/).
