# Supplementary figures and images for: Long non‐coding RNA SNGH7 Is activated by SP1 and exerts oncogenic properties by interacting with EZH2 in ovarian cancer
Source: J Cell Mol Med. 2020 May 18;24(13):7479–89. doi: 10.1111/jcmm.15373 (PMC7339223; doi:10.1111/jcmm.15373)

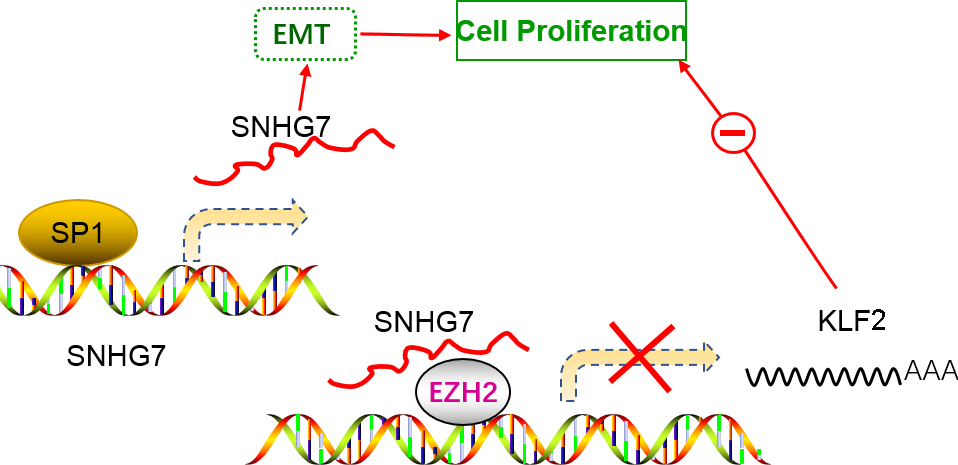

Supplement: Supplementary file 1 — Fig S1 [file JCMM-24-7479-s001.tif]
