# Supplementary material for: Long non‐coding RNA SNGH7 Is activated by SP1 and exerts oncogenic properties by interacting with EZH2 in ovarian cancer
Source: J Cell Mol Med. 2020 May 18;24(13):7479–89. doi: 10.1111/jcmm.15373 (PMC7339223; doi:10.1111/jcmm.15373)
Supplement: Supplementary file 2 — Table S1 [file JCMM-24-7479-s002.docx]

Supplementary Table 1: Sequences of primers for qPCR and siRNA sequence

| \| **Primers for qRT-PCR** \| \| --- \| | Sequences (5′-3′) |
| --- | --- | --- |
| lncRNA SNHG7 (Forward) | AAATGTCAGCAGTGCCAGTG |
| lncRNA SNHG7 (Reverse) | GATGGGGTTTCAGGCAGTTG |
| GAPDH (Forward) | GTCTCCTCTGACTTCAACAGCG |
| GAPDH (Reverse) | ACCACCCTGTTGCTGTAGCCAA |
| KLF2 (Forward) | CTGCACATGAAACGGCACAT |
| KLF2 (Reverse) | CAGTCACAGTTTGGGAGGGG |
| RND1 (Forward) | TGACATCAGCCGTCCAGAGACA |
| RND1 (Reverse) | TGCAGCCAATGAGCAAAACGCG |
| EZH2 (Forward) | TGCACATCCTGACTTCTGTG |
| EZH2 (Reverse) | AAGGGCATTCACCAACTCC |
| PTEN (Forward) | TGGATTCGACTTAGACTTGACCT |
| PTEN (Reverse) | GGTGGGTTATGGTCTTCAAAAGG |
| **Interference sequences (siRNA)** |  |
| siRNA 1# (SNHG7) | GCUGGAAUAAAGAGUAACAUU |
| siRNA 2# (SNHG7) | CCAGAGAACCUGCCUUCCUUCCU |
| si-NC | UUCUCCGAACGUGUCACGUTT |
| si-EZH2 | GAGGUUCAGACGAGCUGAUUU |
